# Assessing the Impact of Isoniazid Preventive Therapy (IPT) on Tuberculosis Incidence and Predictors of Tuberculosis among Adult Patients Enrolled on ART in Nekemte Town, Western Ethiopia: A Retrospective Cohort Study

**DOI:** 10.1155/2019/1413427

**Published:** 2019-05-02

**Authors:** Gemechu Tiruneh, Alemayehu Getahun, Emiru Adeba

**Affiliations:** ^1^Department of Medical Laboratory Sciences, College of Medical and Health Sciences, Wollega University, Nekemte, P.O. Box: 395, Ethiopia; ^2^Department of Public Health, College of Medical and Health Sciences, Wollega University, Nekemte, P.O. Box: 395, Ethiopia

## Abstract

**Background:**

Isoniazid preventive therapy is a prophylactic treatment used in the prevention of active tuberculosis. It is known to be most effective in preventing tuberculosis in patients with positive tuberculin skin test.

**Methods:**

A retrospective cohort study centering on two institutions in Nekemte town, Western Ethiopia, was employed. Secondary data of 600 medical records were analyzed by Cox regression.

**Result:**

Tuberculosis incidence among the Isoniazid treated group was 1.98 per 100 person-years and 4.52 per 100 person-years in the untreated group. CD4 cell count, clinical staging, body mass index (BMI), not using cotrimoxazole, body weight, and functional status were significant predictors of tuberculosis risk. Isoniazid preventive therapy use was associated with 55% reduction of tuberculosis incidence.

**Conclusion:**

Isoniazid preventive therapy use was associated with significant reduction in tuberculosis incidence, even in the absence of Tuberculin Skin Test (TST). Therefore, isoniazid preventive therapy (IPT) coverage should be used more widely, with special emphasis given to patients at higher risk of tuberculosis. The study shows that the absence of TST testing should not be a limitation.

## 1. Background

Tuberculosis (TB) is the commonest of all opportunistic infections in people living with Human Immune Deficiency Virus (HIV) and causes preventable Acquired Immune Deficiency Syndromes (AIDS) related mortality and morbidity, especially in sub-Saharan Africa. The risk of acquiring TB for HIV positive individuals is 26-28 times greater than in HIV negative individuals [1]. The high rate of TB in HIV infected individuals gives rise to the need for strategies to prevent TB in this population. The World Health Organization (WHO) recommends isoniazid preventive therapy (IPT) as part of the three* I's *strategies for TB/HIV coinfection: IPT, intensified TB case finding, and infection control for TB [1, 2]. IPT is a proven strategy for reducing TB in individuals at a community and population level. The effectiveness of IPT in preventing TB has been well established in HIV-negative individuals and communities as well as in HIV-infected populations [2–4]. The greatest benefit of IPT in preventing TB is clearly established for people with confirmed latent TB infection which is diagnosed by a positive TST [5, 6].

In Ethiopia, TST is not applicable to initiate IPT. Therefore, initiation of IPT is based on symptomatic screening to rule out the presence of active TB; this could limit the effectiveness of IPT by missing those with latent TB[6–8]. This study aims to review the effectiveness of IPT in a country like Ethiopia where TST and other techniques used to diagnose latent tuberculosis infections (LTBI) are absent. Adherence to IPT is important and poor adherence will limit its impact. Unlike other studies which include nonadherent patients in their assessment of IPT impact, this study only compares patients who complete a full course of treatment with an untreated group. Furthermore, this study will identify other factors that favor the occurrence of TB which may be useful in identifying areas of future focus in the control and management of TB among patients living with HIV.

## 2. Methods

### 2.1. Study Setting and Context

The study was a facility-based retrospective cohort study, with a three-year followup, using clinical data from adult HIV patients receiving ART/HAART (antiretroviral therapy/highly active antiretroviral therapy) at two health facilities in Nekemte town: Nekemte referral hospital and Nekemte health center. The health facilities are pioneers in providing chronic HIV care services in Ethiopia. A total of 7500 adult HIV patients receive ART across both institutions, of which 5000 are based at Nekemte referral hospital [9].

### 2.2. Study Population

The study was based on secondary data retrieved from clinical registers of patients starting HIV care between 2009 and 2012. The subjects included in the study were selected based on eligibility criteria set for IPT exposed and unexposed cohorts. The cohorts were defined based on whether the patient had received IPT, which was regarded as the primary exposure variable of the cohorts. Patients who had completed six months of isoniazid prophylaxis were classified in the exposed cohort while patients who had never been offered IPT were considered unexposed. Adult patients enrolled on ART, aged 18 and above, who were free of active TB and who had completed six months of Isoniazid therapy were considered eligible candidates and were included in the exposed group. For the unexposed cohort, patients who had never initiated IPT and who met all other previously stated criteria were included.

### 2.3. Sample Size and Sampling Procedure

The required sample size was determined using Epi-info Version 7 assuming an independent cohort study. It was assumed that Isoniazid completers were exposed throughout the followup and untreated groups were not exposed. The incidence of TB amongst IPT treated groups was 4.36% and 12% for non-IPT groups, taken from a former study [8]. Power of 80% and 5% margin of error, with exposed to unexposed ratio of 2:1, was taken into account. Therefore, the final sample size was 400 for unexposed and 200 for the exposed group. Qualified candidates were sorted based on predefined inclusion criteria for both groups. IPT registration logbook was used as a frame to screen the exposed group and the ART registration logbook for the unexposed group. The study subjects were selected for each group independently using a simple random sampling technique where purposive serial numbers were assigned to each of the presorted patient folders. The final study population was selected using a lottery method until the predetermined sample size was attained for each cohort.

### 2.4. Variables and Data Collection Procedures

The primary exposure variable was IPT receipt while incidence of TB was the main outcome variable. Sociodemographic and clinical variables were considered in order to identify predictors of TB incidence during the followup process. Those patients who were lost to followup, study cessation year, 10^th^ of September 2012, died, or who transferred out of area were considered as censorship states during the analysis. In the non-IPT group (unexposed) those who initiated IPT prophylaxis were censored on the date of IPT initiation. Every patient was followed until the occurrence of TB or any of the censorship states which occurred first.

### 2.5. Data Analysis

Data was entered, sorted, double-checked, and analyzed using SPSS version 20. Univariate analysis was performed to show the summary of each variable of the entire cohort using a simple frequency table. To identify predictors of TB incidence, every variable in the bivariate analysis of Cox proportional hazard model with crude hazard ratio of p-value < 0.2 was selected to build the multivariate model. Each variable was checked for confounding effect against IPT and change in regression coefficient of each variable by less than 20% when compared with a crude model revealed absence of confounding. Interaction effect or effect modification was also assessed to check whether the effect of IPT was modified when variables were included in the multivariate model henceforth; all interaction models were statistically insignificant. Universal assumptions of Cox proportional hazard model was tested by graphical methods using log-log plot. The assumption of Cox proportional hazard model was not violated and the result is valid at specified significance level and degree of freedom.

## 3. Results

### 3.1. Baseline Sociodemographics and Clinical Description of the Cohorts

A total cohort of 600 HIV patients on ART, comprising 200 IPT and 400 non-IPT, were followed for a median duration of 26 (IQR 20-33) months. 356 (59 %) of the patients were female. Mean baseline age of the both cohorts was 33 years old with SD 9. Half (51%) were married at baseline, and 106 (18.43%) widowed. The main religious group was Ethiopian Orthodox, comprising 237(41%), and 154 (27%) of the cohorts were educated up to secondary school level (Table 1).

The median CD4 count at baseline for the entire cohort was 381 (IQR 262-535). 517(86.16%) of the cohort had adequate functional status at baseline to perform their daily duties. The majority (76.33%) of the entire cohort were categorized as WHO clinical stage I or II at the start of followup. 60% of the study subjects had a BMI≥ 18.5 at enrollment while 56.3% of the entire cohorts were higher than 50kg at baseline (Table 2).

### 3.2. Incidence of TB

600 study participants were followed for a total of 1482.55 person-years of observation. 53 (8.8%) of participants developed TB while in followup and 547 individuals were censored from the study, giving an overall incidence of TB during the followup period, 3.57 cases per 100 person-years. The TB incidence rate in the IPT group was 1.98 per 100 person-years and 4.52 per 100 person-years for the non-IPT group. Among the TB cases that occurred in the followup period, 34 cases occurred in females giving an incidence in females of 3.85 per 100 person-years and more than half, 31(58.5%), of the TB cases were diagnosed in those under 30 years old with a crude incidence rate of 4.15 cases per 100 person-years in this age group. During the followup period, the highest TB rate was observed in patients with a CD4 counts below 200 cells/ul at baseline, accounting for 12.3 cases per 100 person-years (Table 3).

### 3.3. Predictors of Tuberculosis

After fitting candidate variables into multivariate Cox-regression model, most baseline variables were found to be predictors of TB. PLWHIV (People Living With HIV) at WHO clinical stage III or stage IV had 3.22 times higher risk of acquiring TB compared to those at WHO clinical stage I or II (AHR =3.22, 95%CI = 1.07-9.7). Patients with a baseline CD4 count below 200 cells/ul were fifteen times more likely to develop TB than patients with a CD4 count greater than 500 cells/ul (AHR 15, CI=5.14-43.3). Similarly, PLWHIV with a CD4 count of between 200 and 350 were 4.6 times more likely to have TB compared to patients with a CD4 count greater than 500 cells/ul (AHR 4.57, CI=1.5-13.8). Patients who were not treated with cotrimoxazole had 3.47 increased risk of developing TB during the followup compared to treated counterparts (AHR=3.47, 95%CI 1.88-6.39). Those patients whose body weight was below 50kg were at 1.87 increased risk of developing TB compared to those whose weight was greater than 50kg (AHR=1.87, 95%CI 1.02-3.4). Similarly, patients with a BMI below 18.5kg/m^2^ had a 1.85 greater chance of developing TB than a patient with a BMI above 18.5kg/m^2^ at baseline (AHR= 1.85, CI=1.02-3.55). Patients who were bedridden or whose ambulatory functional status was poor were 2.22 times more likely to develop TB compared to patients who were at working functional status during baseline (AHR= 2.22, CI=1.12-4.41) (Table 4).

### 3.4. Impact of IPT

After controlling for other variables, the overall effect of IPT was found to reduce TB incidence by 55% (AHR= 0.45, CI=0.219- 0.920). In unadjusted bivariate analysis the crude effect of IPT was nearly 60% (UHR= 0.397, CI=0.203- 0.774). Each of the covariate was checked against IPT or IPT was adjusted for each of the covariate independently; none of the covariates altered the effect of IPT by more than 20%. Interaction analysis was also performed to check whether IPT had a differential effect on subgroups of each covariate, but a p-value of the interaction term was statistically insignificant and did not demonstrate any differential effect of IPT on covariates. (Table 5)

## 4. Discussion

In this study, the overall incidence of TB was 3.57 per 100 person-years among patients on ART. This is comparable with other studies done in Ethiopia and India, which reported a TB incidence of 3.73 and 2.83 per 100 person-years, respectively, among those on ART [10, 11]. However, the findings of this study are lower than the findings of the two other studies conducted in Ethiopia which reported 7.0 TB cases per 100 person-years and 8.6 cases per 100 person-years, respectively [12, 13]. However, these studies were not specifically related to patients on ART which may explain the discrepancy. With prolonged ART, the risk of developing TB is minimized [14, 15]. In this study, the incidence of TB was higher among patients not on IPT with an incidence of 4.42 per 100 person-years when compared to patients on IPT prophylaxis who had a TB incidence of 1.98 per 100 person-years. This is in agreement with most of the studies conducted in Ethiopia and other countries with higher TB burden [3, 7, 8, 16–18].

This study has also identified several predictive risk factors for TB among HIV infected people enrolled on ART irrespective of their IPT status. One of the important predictors for TB occurrence was baseline clinical variables like WHO clinical stage. Even though TB can occur at any HIV clinical stage, it is more common in advanced stages. PLWHIV at WHO clinical stage III or IV were at 3.22 times higher risk of acquiring TB compared to those are at WHO clinical stage I or II. Different authors have demonstrated the positive association between TB incidence and advanced stage of HIV and this has also been found in other studies within Ethiopia [12, 14, 19, 20]. The same result is explained in various studies conducted out of Ethiopia concurring with this study [8, 10, 15, 17, 21]. Another important finding associated with incident TB was CD4 count below 200 which was found to be a strong predictor of TB. Patients with CD4 count below 200 cells/ul have more risk of developing TB as compared to patients with CD4 count above 500 cells/ul. Similar result is reported by numbers of researches with varying range of risks [10, 12, 15]. Similarly, PLWHIV with a CD4 count of between 200 and 350 cells/ul were 4.6 times more likely to have TB than a patient with a CD4 count greater than 500 cells/ul. This result was similar to studies done in Ethiopia and which showed there is a two to three times higher risk in patients with lower CD4 counts [12]. Another important finding of this study was not being treated with cotrimoxazole as an independent predictor of TB. Those patients not treated with cotrimoxazole had 3.47 times greater risk of developing TB than untreated groups (AHR=3.47, 95%CI 1.88-6.39). This result can confirm that using cotrimoxazole has added benefit in prevention of TB as evidenced in other researches as well [14, 17, 21]. PLWHIV with a baseline body weight below 50kg were 1.87 times more likely to have TB than a patient with a body weight above 50kg. There are also other studies supporting this finding. For example, individuals with a body weight below 50kg were more likely to have TB and also prone to failing ART in a study. This shows that patients with higher body weight were less likely to develop TB than those with lower body weights [4, 7, 9]. Additionally, TB cases were more likely to occur in patients with a BMI below 18.5. This finding corresponds with a study conducted in northwest Ethiopia which revealed that patients with a BMI of <18.5 at baseline were 1.62 times more likely to get TB as compared to adults with BMI≥18.5 at baseline [9, 10]. One possible explanation for this is that debilitated patients are more prone to malnutrition and less physical activity and consequently are more vulnerable to many diseases, including TB [9].Another prospective study from Tanzania showed that lower BMI and falling BMI in HIV positive patients was a strong predictor of active TB [22].This study did not take into account falling BMI and was limited to baseline data due to its retrospective nature. Patients who were bedridden or who had poor ambulatory functional status are more susceptible to develop TB than patients with working functional status during baseline. This is in line with studies from Ethiopia which revealed that the risk of getting TB would be higher if the patient is in a state of poor ambulatory or bedridden with regard to functional status [12, 21].

In this study IPT prophylaxis has shown to have a protective effect with a reduction in TB incidence of 55%; this aligns to other studies conducted in other regions of Ethiopia and other countries with higher TB prevalence [3, 14, 16, 17]. One study conducted in Jimma, western Ethiopia, found a 50% reduction in TB incidence which was solely attributed to IPT used for HIV positive clients [8]. Similarly, another retrospective study in southern Ethiopia within a comparable study setting found the combined effect of IPT and ART, when started simultaneously, reduced TB incidence by 57% compared to treatment with ART only [19]. Correspondingly, a cross-sectional study conducted in Ethiopia showed IPT prophylaxis combined with cotrimoxazole had a protective benefit against TB with 50% rate of TB attrition [17]. In contrary to all of these results, a placebo randomized controlled trail (RCT) from Kenya in HIV infected children showed no beneficial effect of IPT [4]. This disparity could be explained by the difference in study population when compared to the current study and the retrospective nature of the current study.

## 5. Limitations

The main limitation of this study is the retrospective nature of the cohort. Some important variables like viral load, hemoglobin, adherence status, and ART drug regimen were not included due to problems associated with data inconsistency and incompleteness.

## 6. Conclusion and Recommendations

IPT reduces the risk of TB by 55% in patients on ART. The study found administration is beneficial even in the absence of TST to diagnose latent TB infection. Further research is required to explore IPT resistance and to look at how many latent cases are going untreated in Ethiopia in the absence of TST.

## Figures and Tables

**Table 1:** Baseline sociodemographic characteristics of PLWH at Nekemte referral hospital and Nekemte Health center followed from September 2009-September 2012.

Variables	Category	Cohort Group		Total N (%)
		IPT Cohort n (%)	Non-IPT n (%)	

Age Group (n=600)	18-30	102(51%)	199(49.75%)	301(50.16%)
	31-40	68(34%)	140(35%)	208(34.6)
	41-50	25(12.5%)	44(11%)	69(11.5%)
	>50	5(2.5%)	17(4.25%)	22(3.66%)

Sex (n=600)	Female	113(56.5%)	243(60.8%)	356(59.6%)
	Male	87(43.5%)	157(39.2%)	244(40.7%)

Religion (n=575)	Muslim	37(18.8%)	83(21.33%)	120(20.5%)
	Orthodox	73(37.24%)	164(42.16%)	237(40.5%)
	Protestant	82(41.8%)	130(33.42%)	212(36.24%)
	Others∗	4(2.04%)	12(3.08%)	16(2.73%)

Educational Status (n=580)	No Education	39(20.21%)	89(22.94%)	106(18.43)
	Primary Education	84(43.75%)	160(41.28%)	128(22.07%)
	Secondary Education	59(30.73%)	95(24.48%)	154(26.55%)
	Tertiary Education	10(5.2%)	44(11.34%)	54(9.3%)

Marital Status (n=575)	Married	93(48.19%)	200(52.35%)	293(50.96%)
	Single	39(20.21%)	60(15.70%)	99(17.28%)
	Divorced/Separated	26(13.48%)	51(13.35%)	77(13.39%)
	Widowed/Widower	35(18.13%)	71(18.59%)	106(18.43%)

∗ refers to Catholic or Adventist.

**Table 2:** Baseline clinical, laboratory results, and followup outcome characteristics of PLWH at Nekemte referral hospital and Nekemte Health center followed from September 2009-September 2012.

Variables(n=600)	Category	Cohort group		Total n (%)
		IPT n (%) cohort	Non-IPT cohort n (%)	

CD4	<=200	20(10%)	68(11.33%)	88(14.66%)
	201-350	64(32%)	119(29.75%)	183(30.5%)
	351-500	52(26%)	97(24.25%)	149(24.83%)
	>500	64(32%)	116(29%)	180(30%)

BMI	>=18.5	127(63.5%)	229(57.25%)	356(59.33%)
	<18.5	73(36.5%)	171(42.75%)	244(40.66%)

Weight	<=50	85(42.5%)	177(44.25%)	266(40.66%)
	>50	115(57.5%)	223(55.75%)	338(56.33%)

WHO Stage	I/II	164(82%)	294(73.5%)	458(76.33%)
	III/IV	36(18%)	106(26.5%)	142(23.66%)

Functional Status	Working	169(84.5%)	348(87%)	517(86.16%)
	Ambulatory/Bedridden	29(14.5%)	43(10.75%)	72(18%)

Opportunistic Infection	Yes	45(22.5%)	80(20%)	125(20.83%)
	No	155(77.5%)	320(80%)	475(79.16%)

Cotrimoxazole Treatment	Yes	142(71%)	287(71.75%)	429(71.5%)
	No	58(29%)	113(28.25%)	171(28.5%)

Previous TB	Yes	45(22.5%)	80(20%)	125(20.83%)

	No	155(77.5%)	320(80%)	475(79.16%)

Outcome status	TB	11(5.5%)	42(10.5%)	53(8.83%)
	Censored	189(94.5%)	358(89.5%)	547(91.16%)

Censored: loss to followup, death, transferred out, started IPT (for non-IPT).

**Table 3:** Incidence rate of tuberculosis of PLWH at Nekemte referral hospital and Nekemte Health center followed from September 2009-September 2012.

Variables(n=600)	Category	Incident TB Cases	Person Year	Incidence Rate/100 PYO

Age Group	<=30	31	747	4.15
	31-40	10	522.08	1.9
	41-50	7	166.91	4.19
	>50	5	46.56	10.7

Sex	Female	34	881.16	3.85
	Male	19	601.39	3.15

CD4	<=200	29	235.06	12.3
	201-350	16	394.28	4.05
	351-500	4	380.95	1.05
	>500	4	472.26	0.85

BMI	>=18.5	18	903.38	2.0
	<18.5	35	579.17	6.04

Weight	<=50	36	603.07	5.96
	>50	17	852.48	2.0

WHO Stage	I/II	36	1236.66	2.9
	III/IV	19	245.89	6.9

Functional status	Working	35	1271.03	2.75

	Ambulatory/ Bedridden	18	211.52	8.5

Opportunistic Infection	Yes	10	295.17	3.38
	No	43	1187.38	3.6

Cotrimoxazole Treatment	Yes	20	1064.16	1.88
	No	33	418.39	7.88

Cohort group	IPT	11	554.72	1.98
	Non-IPT	42	927.83	4.52

**Table 4:** Predictors of tuberculosis risk, multivariate analysis by Cox proportional hazard model of PLWHA at Nekemte referral hospital and Nekemte Health center followed from September 2009-September 2012.

Variables (n=600)	Category	Unadjusted HR(95%CI	P-value	Adjusted HR(95%CI	P-value

Age	<=30	Referent		Referent	
	31-40	0.47(0.23-0.95)	0.035∗	0.47(0.23-1.05)	0.06^**#**^
	41-50	1.05(0.46-2.38)	0.92	1.05(0.46-2.38)	0.072^**#**^
	>50	2.9(1.13-7.5)	0.027∗	2.9(1.13-7.5)	0.08^**#**^

CD4 Count	>500	Referent			
	351-500	1.25(0.314-5.16)	0.749	1.25(0.89 -5.16)	0.68^**#**^
	201-350	4.9(1.6-14.6)	0.005∗	4.9(1.6-14.6)	0.007
	<=200	17.5 (6.15-50)	<0.001∗	17.5 (6.15-50)	<0.001

Sex	Male	Referent			
	Female	1.095(0.83-	0.525		

BMI	>=18.5	Referent		referent	
	<18.5	3.13(1.77-5.53)	<0.001∗	3.13(1.77-5.53)	0.042

Weight	>50	Referent		Referent	
	<=50	1.72(1.28 -2.28)	0.001∗	1.72(1.28 -2.28)	0.041

WHO Stage	I/II	Referent		Referent	
	III/IV	3.22(0.96-4.56)	0.08∗	3.22(1.96-4.56)	0.037

Functional Status	Working	Referent		Referent	
	Ambulatory/Bedridden	3.38(1.9-5.96)	<0.001∗	3.38(1.9-5.96)	0.022

Cotrimoxazole Treatment	Yes	Referent		Referent	
	No	4.21(2.42-7.4)	<0.001∗	4.21(2.42-7.4)	<0.001

Previous TB	No	Referent			
	Yes	1.43(0.56-3.58)	0.45		

Opportunistic Infection	No	Referent		Referent	
	Yes	1.02(0.51-2.02)	0.06∗	1.02(0.51-2.02)	0.061^#^

Cohort Group	Yes	Referent		Referent	
	No	2.5(1.3-4.9)	.007∗	2.5(1.3-4.9)	0.03

^∗^All candidate variables for multivariate analysis.

^#^Lost statistical significance in multivariate analysis.

**Table 5:** Effect of IPT on reduction of tuberculosis incidence among cohorts of PLWHA at Nekemte referral hospital and Nekemte Health center followed from September 2009-September 2012.

IPT adjusted covariates	Unadjusted hazard 95%CI	p-value	Adjusted Hazard 95 %CI	p-value	Significant reduction in TB (%)	p-value for interaction

IPT	0.397(0.203- 0.774)	0.007	0.449(0.219-0.920)^¥^	0.029	55^¥^	

IPT∗ Cotrimoxazole			0.402(0.206-0.784)	0.007	60	0.219

IPT∗ WHO stage			0.382(0.196-0.745)	0.005	62	0.48

IPT∗ CD4 count			0.348(0.176-0.685)	0.002	65	0.64

IPT∗ Functional status			0.386(0.198-0.754)	0.005	61	0.08

IPT∗ BMI			0.403(0.206-0.788)	0.012	60	0.072

IPT∗ Weight			0.426(0.218-0.831)	0.008	57	0.74

IPT∗ Sex			0.399(0.204-0.778)	0.007	40	0.85

IPT∗ opportunistic infection			0.396(0.203-0.773)	0.007	60	0.61

IPT∗ age			0.403(0.206-0.788)	0.008	60	0.065

IPT∗ previous TB			0.401(0.205-0.785)	0.008	60	0.588

^∗^IPT adjusted for each covariate.

^¥^Overall effect of IPT adjusted for all covariates.

## Data Availability

The data set generated from patients' clinical record is not publicly available to protect patient confidentiality. Unidentifiable data can be obtained from the corresponding author upon reasonable request.

## List of Abbreviations

AHR: Adjusted hazard ratio
AIDS: Acquired immune deficiency syndrome
ART: Antiretroviral therapy
BMI: Body mass index
CI: Confidence interval
HAART: Highly active antiretroviral therapy
HIV: Human immunodeficiency virus
HR: Hazard ratio
IPT: Isoniaziad preventive therapy
LTBI: Latent tuberculosis infection
PLHIV: People living with human immune deficiency virus
OI: Opportunistic infection
SPSS: Statistical package for social science
TST: Tuberculin skin test
TB: Tuberculosis.
